# Genome-Wide Identification of GAST Family Members and Their Potential Roles in Epicotyl Dormancy in Chinese Cork Oak (*Quercus variabilis*)

**DOI:** 10.3390/plants13091247

**Published:** 2024-04-30

**Authors:** Yaochen Wang, Yifei Huang, Yixin Chen, Zhaowei Yu, Puyuan Liu, Guolei Li, Qinsong Yang

**Affiliations:** 1 Research Center of Deciduous Oaks, Beijing Forestry University, Beijing 100083, China; 18829072322@163.com (Y.W.); 18950679107@163.com (Y.H.); chenyixin@bjfu.edu.cn (Y.C.); yzw23583145@bjfu.edu.cn (Z.Y.); puyuan622531@bjfu.edu.cn (P.L.); glli226@163.com (G.L.); 2 Deciduous Oak Improvement and Regeneration Innovation Team of State Forestry and Grassland Administration, Beijing Forestry University, Beijing 100083, China; 3 Key Laboratory for Silviculture and Conservation, Ministry of Education, Beijing Forestry University, Beijing 100083, China

**Keywords:** Chinese cork oak, *GAST* gene family, epicotyl dormancy, gibberellin, abscisic acid

## Abstract

Chinese cork oak (*Quercus variabilis* Blume) is a widespread tree species with high economic and ecological values. Chinese cork oak exhibits epicotyl dormancy, causing emergence heterogeneity and affecting the quality of seedling cultivation. Gibberellic acid-stimulated transcript (GAST) is a plant-specific protein family that plays a crucial regulatory role in plant growth, development, and seed germination. However, their evolution in Chinese cork oak and roles in epicotyl dormancy are still unclear. Here, a genome-wide identification of the *GAST* gene family was conducted in Chinese cork oak. Ten *QvGAST* genes were identified, and nine of them were expressed in seed. The physicochemical properties and promoter cis-acting elements of the selected Chinese cork oak *GAST* family genes indicated that the cis-acting elements in the *GAST* promoter are involved in plant development, hormone response, and stress response. Germinated seeds were subjected to gibberellins (GAs), abscisic acid (ABA), and fluridone treatments to show their response during epicotyl dormancy release. Significant changes in the expression of certain *QvGAST* genes were observed under different hormone treatments. *QvGAST1*, *QvGAST2*, *QvGAST3*, and *QvGAST6* exhibited upregulation in response to gibberellin. *QvGAST2* was markedly upregulated during the release of epicotyl dormancy in response to GA. These findings suggested that *QvGAST2* might play an important role in epicotyl dormancy release. This study provides a basis for further analysis of the mechanisms underlying the alleviation of epicotyl dormancy in Chinese cork oak by *QvGASTs* genes.

## 1. Introduction

Epicotyl dormancy is a survival strategy in plants that serves as an adaptation to the environment. It provides a mechanism for delaying germination, assisting plant seeds in overcoming adverse conditions, such as freezing and drought, until the conditions become more favorable for the survival of the next generation [1]. The breaking of epicotyl dormancy depends on the levels of gibberellins (GAs) and abscisic acid (ABA) in the hypocotyl of germinating seeds [2]. This process is often accompanied by an increase in internal GAs and a decrease in ABA [3,4,5]. Similar effects have been observed in other plants: Arabidopsis seeds treated with ABA and GAs exhibit significant differences in the content of intracellular microtubules, which is related to the mechanism of breaking epicotyl dormancy [6]. Exogenous GA_3_ treatment breaks the epicotyl dormancy of peony seeds [7]. Effective relief of epicotyl dormancy in peony by low-temperature treatment is associated with a significant increase in endogenous GAs [2]. In the process of relieving epicotyl dormancy in *Polygonatum sibiricum* seeds, the biosynthesis and signal transduction pathways of GA are detected [8]. In the case of *Paeonia emodi* Wall. Ex Royle., the ABA/GA_3_ ratio gradually decreases during the relief of epicotyl dormancy by cold stratification, and starch content is significantly correlated with the activities of α-amylase and β-amylase [9]. Therefore, we speculated that GAs and ABA might be related in the process of relieving epicotyl dormancy.

The *GAST* (GA-stimulated transcripts) gene family, which is referred to as the *GASA* (gibberellic acid stimulated in Arabidopsis) gene family in certain plants, is widely distributed in the plant kingdom and is characterized by a C-terminal region consisting of approximately 60 amino acids, containing 12 conserved cysteine residues at specific positions, referred to as the *GAST* domain [10]. Cysteine residues are essential for forming disulfide bonds, which play a crucial role in protein folding and interactions between proteins. This region is critical for maintaining the spatial structure and function of GAST proteins [10,11]. The first identified member of the *GAST* family in tomatoes (*Solanum lycopersicum*) was called gibberellin-stimulated transcript 1 (*GAST*-1) [12]. As different species’ *GAST* gene family members have been identified, comprehensive insights into the functions of this gene family have been gained, including in *Arabidopsis thaliana* [13], *Oryza sativa* [14], *Brassica rapa* [15], *Phyllostachys edulis* [16], *Phaseolus vulgaris* [17], *Pyrus pyrifolia* [1], *Prunus mume* [18], *Cucumis sativus* [19], and *Paeonia ostia* [7]. *GAST* genes participate in various plant growth and development processes, including seed germination, flower induction, and stem elongation, playing crucial regulatory roles [20]. In Arabidopsis, most *GAST* genes are strongly expressed in the abscission zones of flowers and siliques [13]. Peanut *AhGASA1* and *AhGASA18* show higher expression levels in large seeds at various stages of pod development compared to small seeds, suggesting their potential importance in pod development [21]. The *GAST* gene family is also involved in the regulation of plant growth, development, and stress responses. Due to the conserved structure of the *GAST* domain, which includes 12 conserved cysteine residues, GAST proteins may play a role in plant defense responses [13]. Cotton *GhGAST4* and *GhGAST18* are strongly induced under low-temperature conditions and effectively enhance cotton cold resistance [17]. However, different *GAST* members in Arabidopsis may have opposing effects on development. For example, *AtGASA4* exhibits a promoting effect on flower meristem, while At*GASA*5 shows an inhibitory effect [22]. Considering that the *GAST* gene family could be extensively involved in regulating the plant hormone signaling transduction network, GAST proteins also influence plant hormone responses, primarily regulated by gibberellins, and also participate in the regulation of abscisic acid and other plant hormones. Despite the significant role of *GAST* genes in plant growth regulation, whether they are involved in seed epicotyl dormancy release is still unknown.

Chinese cork oak (*Quercus variabilis*), widely distributed in East Asia [23], is a key species and resource in the formation of deciduous broad-leaved forests in the warm temperate zone and deciduous, evergreen broad-leaved forests in the northern subtropical zone [24]. It holds significant ecological and economic value [25,26,27]. Due to various limitations in asexual reproduction, oak species heavily rely on seeds for propagation [26]. Under natural conditions, Chinese cork oak seeds exhibit epicotyl dormancy; after maturing and falling in autumn, the hypocotyl rapidly germinates and forms roots to prevent water loss, maintaining seed vitality. The epicotyl axis enters dormancy during the autumn and winter seasons and sprouts in the following spring, protecting the seedlings from freezing. In artificial seedling cultivation, the requirement for rapid and uniform germination poses a challenge, as the depth of epicotyl dormancy varies among seeds from the same batch, resulting in differences in germination times ranging from several weeks to even two months [28]. This discrepancy leads to uneven seedling quality, prolongs the seedling management period, and increases labor costs [29]. In the context of Chinese cork oak research, there is a lack of information regarding the *GAST* gene family. Overall, research on the seed dormancy of Chinese cork oak is still in its infancy and currently lacks genes of significant utility. Therefore, understanding the mechanisms underlying the relief of epicotyl dormancy in Chinese cork oak is crucial for seedling cultivation.

Here, we identified 10 *GAST* family genes from the genome of Chinese cork oak and speculated on the possible functions of some *QvGAST* genes through bioinformatics and transcriptome analysis. Additionally, to further validate the mechanisms of action of *QvGAST* genes in response to hormones, we conducted qRT-PCR analysis after different hormone treatments, exploring the regulatory functions of the *GAST* gene family in the process of relieving seed dormancy in Chinese cork oak seeds. To our knowledge, this study marks the inaugural thorough examination of *GAST* genes within the Chinese cork oak, providing an exhaustive assessment. Furthermore, there are few reports on the involvement of this gene family in seed dormancy. This research holds significant implications for the breeding and genetic improvement of Chinese cork oak, providing theoretical references for the seedling cultivation process of Chinese cork oak.

## 2. Materials and Methods

### 2.1. Plant Material

The Chinese cork oak (*Quercus variabilis*) seeds used in this study were collected from Chuzhou, Anhui, in 2023.

### 2.2. Exogenous Treatments of Different Plant Growth Regulators

Before exogenous treatments were applied, the seeds underwent 60 days of cold stratification, followed by 3 days of germination at 21 °C in an incubator. The germinated seeds (in epicotyl dormancy stage) were subjected to treatments with GA_4+7_, ABA, fluridone (FLU, an ABA biosynthesis inhibitor), and 0.02% ethanol (solvent for hormone stock solution as a control). After 12 h soak, the seeds were sowed and cultured under comfortable conditions (24 °C, with a daily light cycle of 16 h light/8 h dark, and light intensity of 5000 lx.). Subsequently, samples were taken after 12 h and 24 h of sowing and immediately frozen in liquid nitrogen for further qRT-PCR validation.

### 2.3. Physicochemical Property Analysis of QvGAST Gene Family Members

#### 2.3.1. Identification of *QvGAST* Genes

The Chinese cork oak genome was assembled by our research group (https://figshare.com/s/67f664d5f6603982128a, accessed on 27 November 2023), and the Hidden Markov Model (HMM) file for the conserved domain of the *GAST* gene family was downloaded from the Pfam database (https://pfam.xfam.org/, accessed on 27 November 2023), with protein accession number PF02704. Protein sequences of Arabidopsis *GASA* family genes were aligned to protein sequences of Chinese cork oak. The HMMER3 3.3.2 software (http://hmmer.org) was employed for searching the entire Chinese cork oak genome protein sequences, using the GAST HMM domain as the query condition. Simultaneously, amino acid sequences of Arabidopsis *GASA* gene family members were downloaded from TAIR11 (https://www.arabidopsis.org/, accessed on 30 November 2023). These sequences were used as query sequences, and the Chinese cork oak genome protein sequences were used as library sequences for BLAST comparison with an E-value set to 1 × 10^−5^. The combined results from both methods were used to finalize the selection of 10 *QvGAST* family genes. The TBtools software (https://github.com/CJ-Chen/TBtools/releases, accessed on 30 November 2023) [30], along with the Gene Location plugin, was utilized to obtain the relative chromosomal positions and gene density information of the target genes on each chromosome based on Chinese cork oak genome annotation data. Chromosome distribution was visualized accordingly. The TBtools software, specifically the Protein Parameter Calc plugin, was used for the physicochemical property analysis of the Chinese cork oak *GAST* gene family. The amino acid sequences of the Chinese cork oak *GAST* gene family were subjected to structure prediction using the SWISS-MODEL online platform (https://swissmodel.expasy.org/, accessed on 23 December 2023). Multiple results were obtained and evaluated based on GMQE (Global Model Quality Estimation) and QMEAN (Qualitative Model Energy Analysis) scores. GMQE ranges from 0 to 1, with higher values indicating better quality, while QMEAN ranges from −4 to 0, with values closer to 0 indicating better alignment with template proteins. The best model was selected based on these criteria, and the predicted protein structure was obtained.

#### 2.3.2. Evolutionary Analysis of *QvGAST* Gene Family

To construct the evolutionary tree, the MEGA 7.0 software was employed. The full-length protein sequences of 15 known Arabidopsis *GASA* gene family members, wheat TaGASR7 and TaGASR34, rice OsGSR1, and pear PpyGAST1 [1,13,31,32] and the 10 Chinese cork oak *GAST* gene family members were used. The parameters were set as follows: Neighbor-Joining (NJ) method, 1000 bootstrap replicates, Poisson model, and pairwise deletion. The classification of subfamilies for the Chinese cork oak *GAST* gene family members was performed based on the analysis method used for the published Arabidopsis *GASA* gene family. The obtained evolutionary tree was further enhanced for visualization using the Chiplot website (https://www.chiplot.online/#, accessed on 7 January 2024).

#### 2.3.3. Gene Structure and Protein Motif Analysis of QvGASTs

For motif analysis, the MEME website (https://meme-suite.org/meme/tools/meme, accessed on 8 January 2024) was utilized. The identification of 6 motifs was set as the criterion, and the results were visualized using the TBtools software for protein-conserved motif analysis. The annotation file of the Chinese cork oak genome and the IDs of the Chinese cork oak *GAST* gene family members were uploaded to the TBtools software to visualize the gene structures.

#### 2.3.4. Cis-Element Analysis of the Promoters of *QvGASTs*

The upstream 2000 bp sequences of the *QvGAST* gene CDS were extracted using the TBtools software. PlantCARE website (http://bioinformatics.psb.ugent.be/, accessed on 7 January 2024) was then employed to screen for cis-elements in the *QvGAST* promoter. Visualization of the results was performed using the TBtools software.

### 2.4. RNA Extraction

RNA extraction was carried out using the modified CTAB (cetyltrimethylammonium bromide) method [33]. Total RNA extracted from the previously treated Chinese cork oak seed samples was used for subsequent experiments.

### 2.5. Gene Expression Analysis

Initially, the HiScript II Q RT SuperMix for qPCR kit was used to synthesize cDNA from 1 μg of RNA following the operating manual as previously described [34]. Quantitative primers for the Chinese cork oak *GAST* gene family were designed using the Primer3.0 online tool (http://www.primer3plus.com, accessed on 8 December 2023). The primers are listed in Table S2. Real-time fluorescence quantitative PCR (qRT-PCR) experiments were conducted using the 7300 Real-Time PCR System (Applied Biosystems Company, Waltham, MA, USA). Data were statistically analyzed, and *QvActin7* gene was used as an internal reference for normalization according to our transcriptome data and previous studies [34,35]. The 2^−ΔΔCt^ method was employed to calculate the relative expression levels of the target genes. Statistical analyses were carried out using Sigmaplot 12.5 software based on the two-way ANOVA method, and the least significant difference test of Fisher at 0.05 significant levels was considered. All experiments were performed with four biological replicates.

### 2.6. Tissue-Specific Expression of QvGAST Genes

Transcriptome data from different tissues of Chinese cork oak, which were generated in our previous study [34], were analyzed to obtain the expression patterns of *GAST* genes in various tissues. The transcriptome data of the 10 *GAST* gene family members in different organs of Chinese cork oak were normalized and row-standardized using the HeatMap plugin in the TBtools software [36] for visualization.

## 3. Results

### 3.1. Genome-Wide Identification of QvGAST Genes

**Figure 1:**
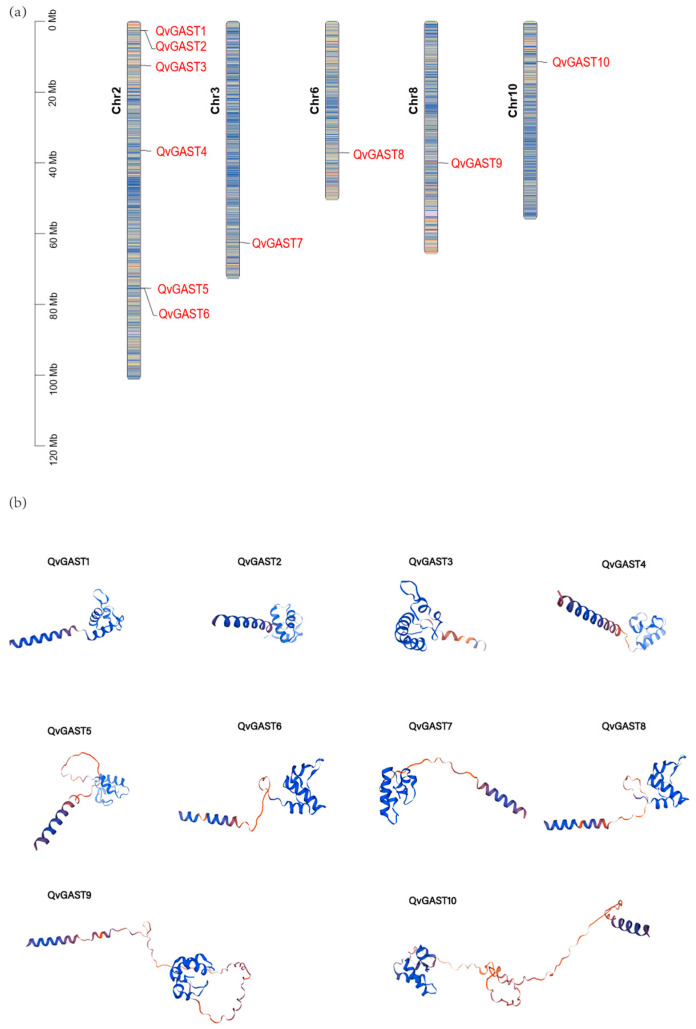
(**a**) The distribution of *GAST* in Chinese cork oak. Color blocks from blue to red indicate a gradual increase in gene density. (**b**) Predicted models of protein structure of QvGAST proteins in Chinese cork oak. The blue color represents regions where the prediction results are deemed highly reliable, while the red color indicates regions with lower reliability in the prediction results.

A total of 10 *QvGAST* genes were identified in our recently assembled Chinese cork oak genome. Chromosomal localization analysis revealed that the 10 Chinese cork oak *GAST* genes were located on chromosomes 2, 3, 6, 8, and 10 (Figure 1a). Specifically, six *QvGAST* genes were found on chromosome 2, whereas chromosomes 3, 6, 8, and 10 each contained one *QvGAST* gene. The 10 *QvGAST* genes were renamed according to their order on the chromosomes, designated as *QvGAST1* to *QvGAST10*.

Physicochemical property analysis (Table S1) showed that the length of the amino acid sequences encoded by Chinese cork oak *GAST* genes ranged from 88 aa (*QvGAST1*, *QvGAST2*, *QvGAST9*) to 194 aa (*QvGAST5*). The isoelectric point ranged from 8.42 (*QvGAST9*) to 9.59 (*QvGAST3*), and the molecular weight ranged from 9.62 kDa (*QvGAST9*) to 20.73 kDa (*QvGAST5*). The instability index of Chinese cork oak GAST proteins ranged from 28.90 (*QvGAST8*) to 91.75 (*QvGAST6*), the aliphatic index ranged from 45.57 (*QvGAST9*) to 80.00 (*QvGAST3*), and the average hydrophilicity coefficient ranged from −0.602 (*QvGAST6*) to 0.110 (*QvGAST3*).

Through the prediction of the protein structure of the Chinese cork oak *GAST* gene family, we found that the protein structure is mainly composed of random coils and α-helices but also includes β-folded structures (Figure 1b). It is worth noting that, compared to GAST proteins in other species, such as common beans and Chinese cabbage, the GAST proteins in Chinese cork oak also exhibit similar structural features [15,37].

### 3.2. Evolutionary Analysis of QvGAST Gene Family

**Figure 2:**
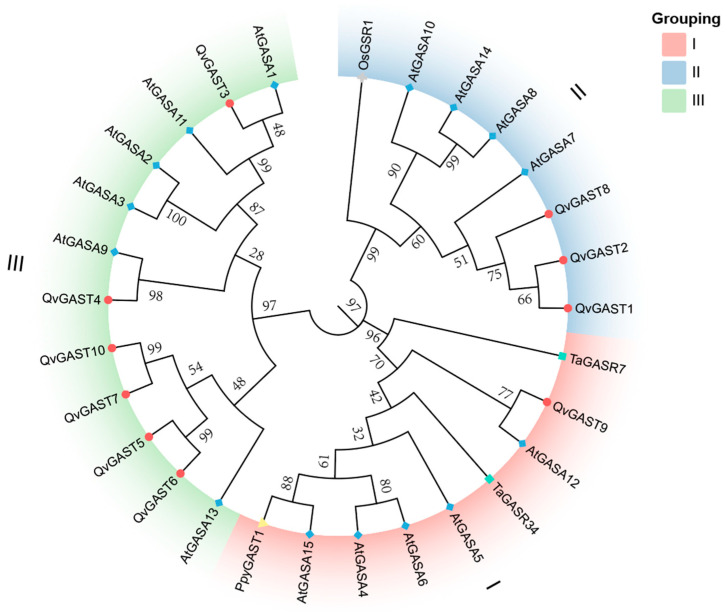
Evolutionary analysis of 29 *GAST* family genes from *Quercus variabilis*, *Arabidopsis thaliana*, *Oryza sativa*, *Triticum aestivum,* and *Pyrus pyrifolia*. A Neighbor-Joining evolutionary tree was constructed, substantiated by 1000 bootstrap iterations in MEGA. The GAST proteins were differentiated into three distinct groups (I, II, III), each designated with a unique color.

To gain further insights into the evolutionary relationship among these *GAST* genes in Chinese cork oak and other species, we constructed an evolutionary tree using the identified 10 *QvGAST* protein sequences, along with 15 full-length Arabidopsis GASA protein sequences and other reported GAST proteins. Following the classification of the Arabidopsis *GAST* gene family, the Chinese cork oak *GAST* family members were divided into three subfamilies, namely Classes I–III (Figure 2). Class I had one member, Class II had three members, and Class III had the largest number of members, with six members.

### 3.3. Gene Structure and Protein Motif Analysis of QvGAST Genes

**Figure 3:**
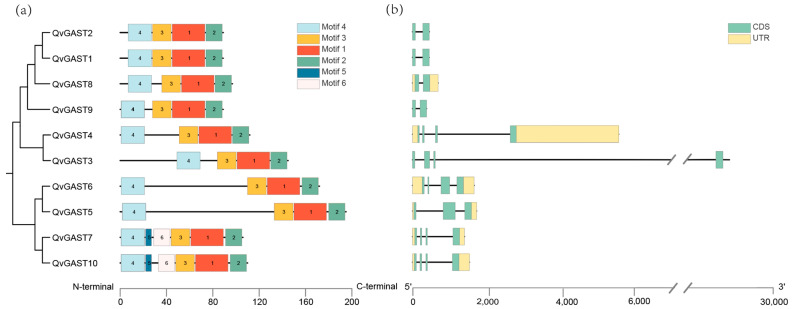
Examination of protein motifs and gene architectures for the *GAST* family genes discovered within the Chinese cork oak. (**a**) A dendrogram depicting QvGAST protein sequences, featuring conserved motifs in distinct colorations. (**b**) Exon–intron distribution analysis of *QvGAST* genes. The green boxes represent CDS, the green boxes represent UTR, and the black lines represent intron positions, respectively.

To analyze the conservation of the Chinese cork oak *GAST* gene family protein sequences, we visualized the gene structures. We analyzed six conserved motifs in the protein products. As shown in Figure 3a, the protein sequences of this family are relatively conserved, with Motif 1-4 universally present in the motifs and distributed in a highly consistent arrangement. Overall, the results indicate that protein sequences in the same clustering branch share similar conserved motifs, except for *QvGAST5* and *QvGAST6*. However, there are differences in motif distribution and quantity between individual genes. For example, *QvGAST7* and *QvGAST10* have Motif 5 and Motif 6 with a highly similar arrangement, suggesting they may have a specific shared function.

To explore the diversity of the Chinese cork oak *GAST* gene structures, we conducted a gene structure analysis. The results (Figure 3b) show significant differences in the numbers of exons and introns among the 10 Chinese cork oak *GAST* genes. In general, genes clustered together mostly have similar gene structures, indicating potential shared biological functions. All *QvGAST* genes have introns, and the gene structures vary, with exon numbers ranging from 2 to 4 and intron numbers from 1 to 3. Specifically, *QvGAST4* has a longer UTR at the 3′ end, and *QvGAST3* has a significantly different intron length compared to other genes.

### 3.4. Cis-Acting Element Analysis of QvGAST Genes

**Figure 4:**
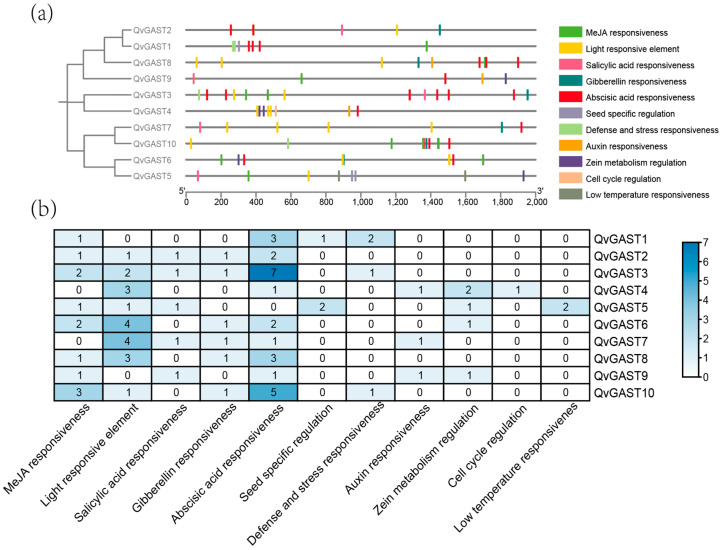
Cis-acting element distribution of *QvGAST* gene family in Chinese cork oak. (**a**) Identification of cis-elements present in the promoter regions of *QvGAST* genes. (**b**) The figures and colors represent the quantity of cis-elements cataloged in the promoters of individual *QvGAST* genes.

*GAST* genes are widely involved in plant hormone signaling pathways and responses to abiotic stress. To understand the potential biological functions of *QvGAST* genes, we analyzed the cis-acting elements in the promoter regions of the Chinese cork oak *GAST* gene family, identifying 11 important cis-acting elements (Figure 4). These elements are mainly associated with hormone response, environmental stimuli, abiotic stress, and stress response.

The results revealed that, except for *QvGAST5*, nine genes have abscisic acid (ABA) response elements, and eight genes, excluding *QvGAST5* and *QvGAST7*, have methyl jasmonate (MeJA) response elements. *QvGAST2*, *QvGAST3*, *QvGAST7*, *QvGAST8*, and *QvGAST10* show GA response elements. Simultaneously, there are elements responsive to salicylic acid, auxin, zeatin, and other hormones, indicating a close association between Chinese cork oak *GAST* genes and hormone signal regulation. Regarding stress-related response elements, *QvGAST5* contains low-temperature stress response cis-elements, suggesting its potential role in cold stress resistance. *QvGAST1*, *QvGAST3*, and *QvGAST10* contain defense and stress response elements, indicating that these Chinese cork oak *GAST* genes may possess abilities to resist adversity and respond to stress. Additionally, light response elements are widely distributed in the *GAST* family, suggesting that *GAST* genes may be regulated by light signals. In summary, the *QvGAST* family participates in crucial physiological processes during Chinese cork oak growth and development.

### 3.5. Tissue-Specific Expression of QvGAST Genes

**Figure 5:**
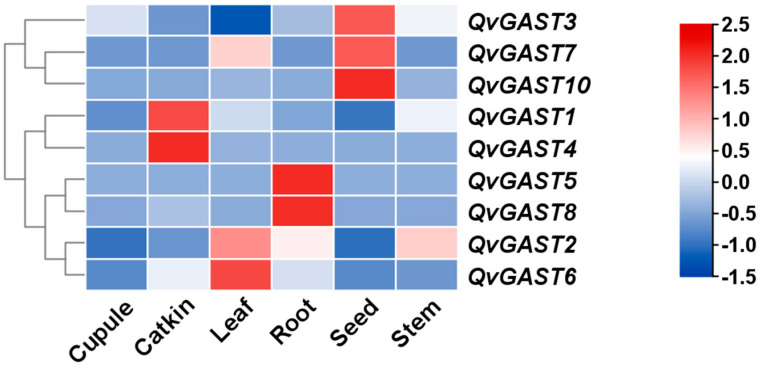
Expression patterns of *GAST* family members in different organs of *Q. variabilis*. Red blocks represent high levels of TPM values, blue blocks represent low levels of TPM values, and white blocks represent the middle level of TPM values after normalization.

To further understand the regulatory mechanisms of the Chinese cork oak *GAST* gene family in relieving seed dormancy and the response of Chinese cork oak seeds to GA, we utilized transcriptome data from various Chinese cork oak organ samples to analyze the gene expression levels of *QvGAST* family members. The transcriptome data were log-transformed, row-standardized, and used to generate a heatmap with clustering (Figure 5). Through the combination of the heatmap and TPM values from the transcriptome data, we identified transcription factors that are highly expressed in seed tissues and have relatively large TPM values in seeds. As a result, three transcription factors, namely *QvGAST3*, *QvGAST7*, and *QvGAST10*, were preliminarily screened. This analysis suggests that these three Chinese cork oak *GAST* genes may play crucial roles in regulating seed dormancy and responding to gibberellin, providing valuable insights into the molecular mechanisms underlying Chinese cork oak seed development and dormancy release.

### 3.6. Hormonal Regulation of QvGAST Genes during Epicotyl Domancy Release

**Figure 6:**
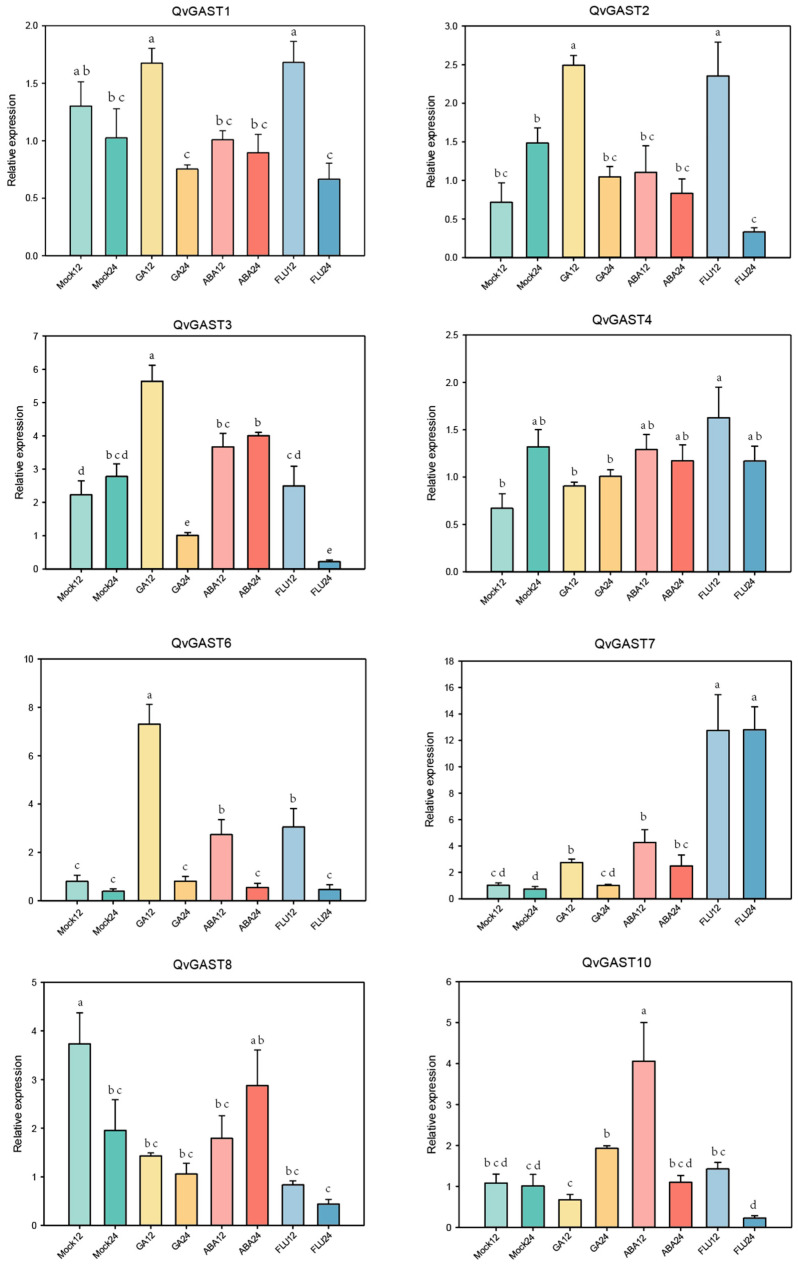
Relative expression levels of *GAST* genes after GA_4+7_, ABA, and FLU treatments at different time points; Mock represents the control group without any hormone treatment. The horizontal axis label “12” represents 12 h after sowing, and “24” represents 24 h after sowing. The error bars indicate the standard deviation among the four biological replicates. Different letters above the column indicate significant differences among the samples (One-way ANOVA analysis with Duncan’s test, *p* < 0.05).

The responsive nature of *GAST* genes to gibberellin (GA) and abscisic acid (ABA) in other species during the process of seed dormancy release and bud dormancy release has been revealed [1,38]. To further characterize the response of *QvGASTs* to GA and ABA during epicotyl dormancy release, the germinated seeds were treated with GA_4+7_, ABA, and FLU (a biosynthesis inhibitor of ABA) at different time points. We measured the expression patterns of *QvGAST* genes to further investigate the potential roles of hormones in Chinese cork oak seed dormancy release (Figure 6).

Two *GAST* genes, *QvGAST5* and *QvGAST9*, did not show expression during this process. The results revealed that GA_4+7_ treatment slightly upregulated the expression of *QvGAST1* and strongly induced *QvGAST2*, *QvGAST3*, and *QvGAST6* within 12 h. ABA treatment led to a slight downregulation of *QvGAST1*, *QvGAST2*, and *QvGAST3*, a slight upregulation of *QvGAST4* and *QvGAST7*, and a strong induction of *QvGAST10* within 12 h. FLU treatment slightly upregulated *QvGAST1* and *QvGAST4* and significantly increased the expression of *QvGAST2* and *QvGAST7*.The differential responses induced by GA and ABA suggest potential antagonistic roles in seed dormancy release and germination processes [33].

Importantly, the expression of these genes varied with the duration of hormone treatment. *QvGAST1*, *QvGAST2*, *QvGAST3*, *QvGAST6*, and *QvGAST7* exhibited a significant upregulation in expression after 12 h of GA_4+7_ treatment, followed by a sharp decrease after 24 h, suggesting a feedback regulation of GA signal. Similar expression patterns were observed for *QvGAST1*, *QvGAST2*, *QvGAST3*, *QvGAST6*, *QvGAST8*, and *QvGAST10* after FLU treatment. *QvGAST10* under GA_4+7_ and ABA treatment and *QvGAST7* under FLU treatment showed a significant upregulation after 24 h. Gene expression changes over time have been observed in studies on *GAST* genes in other species. Notably, the expression of *QvGAST2* and *QvGAST4* was significantly upregulated after 24 h (One-tailed Student’s *t*-test, *p* < 0.05). Additionally, the expression of *QvGAST2* was induced by gibberellin (GA), while it remained unchanged after 24 h of abscisic acid (ABA) treatment, indicating that QvGAST2 was GA responsive and ABA inhibited its expression during epicotyl dormancy release. Consequently, we speculated that *QvGAST2* might be implicated in the regulatory mechanisms governing epicotyl dormancy in Chinese cork oak seeds.

## 4. Discussion

Chinese cork oak (*Quercus variabilis*) is a crucial forestry resource with diverse economic and ecological values. Exploring the molecular mechanisms of epicotyl dormancy release in cork oak is of significant importance in ecology and in artificial nurseries [39,40,41]. Gibberellin (GA) is a class of plant hormones involved in various essential plant developmental processes, including seed dormancy release. *GAST*, a multi-gene family regulated by gibberellins, encodes small peptides rich in cysteine and participates in regulating plant growth and development. Through interactions with plant hormones such as gibberellin and other hormones, *GAST* coordinates the plant’s growth regulatory network.

*GAST* gene family members are identified across numerous plant species, fulfilling vital functions in plant growth and developmental processes [15,21,42]. Although the *GAST* genes have been extensively investigated in diverse plant species, their functional elucidation in perennial woody species is limited, especially during the process of seed epicotyl dormancy release. Considering the significance of the *GAST* gene family, we conducted a comprehensive study on the evolutionary relationships, gene structures, physicochemical properties of proteins, and promoter cis-elements of the cork oak *GAST* gene family.

### 4.1. Genome-Wide Identification and Characteristics of GAST Gene Families in Chinese Cork Oak

This study identified 10 members of the *GAST* gene family in the Chinese cork oak genome. This is fewer compared to the number of *GAST* genes identified in other species, such as *Arabidopsis thaliana* with 15 [13] and *Prunus mume* with 16 [18], suggesting a possible contraction of the gene family in Chinese cork oak. Various characteristics of the GAST protein family were examined in this study, including the number of exons and introns, isoelectric point, and molecular weight. Low molecular weight proteins consistent with other plants were found among QvGASTs. The conserved amino acid sequence at the C-terminus retains all 12 cysteine residues, which showed the characteristic of GAST proteins. QvGAST proteins were found to possess similar structures and motifs, resembling earlier findings in other plants [12,18]. Further investigation revealed that most genes belonging to the same clade had similar exon–intron compositions, suggesting a correlation between the genetic makeup of the GAST domain and its evolutionary past. The distribution of *GAST* genes was irregular, with 10 *QvGAST* genes unevenly distributed across five Chinese cork oak chromosomes (Figure 1a), while no copies of *QvGAST* genes were found on the remaining seven chromosomes. Similar results were observed in peanut, bamboo, and plum [16,18,21].

We analyzed the evolutionary relationships among *QvGAST* genes. Initially, an evolutionary tree of GAST protein sequences in Chinese cork oak was constructed (Figure 2). According to the evolutionary analysis of Arabidopsis *GASA* genes, the identified *AhGASA* genes were classified into three subgroups (I–III). Evolutionarily, we found that the structure of *QvGAST* genes was closely related to their phylogeny. Two genes comprising most gene pairs were shown to have the same motif composition, indicating comparable functionality at the protein level. Furthermore, most AtGASA proteins exhibited conserved domains Motif1, Motif2, Motif3, and Motif4, which were shared among all members of the *QvGAST* gene family. This implies that the particular roles of the *QvGAST* gene family can be inferred from the conservation of genes constituting each branch within the *QvGAST* gene family.

### 4.2. Expression Patterns and Potential Functions of QvGAST Genes

Through tissue-specific expression analysis of *QvGAST* genes, significant differences in expression levels were observed among different genes in cupule, catkin, leaf, root, seed, and stem (Figure 5). Combined with heatmap analysis and transcriptome data in terms of TPM values, we found that *QvGAST3*, *QvGAST7*, and *QvGAST10* were highly expressed in seeds; *QvGAST1* and *QvGAST4* showed relatively high expression levels in catkins; *QvGAST5* and *QvGAST8* were predominantly transcribed in roots. *QvGAST2* and *QvGAST6* exhibited relatively higher expression in leaves. Notably, the transcript of QvGAST9 was undetectable in all six examined plant organs. The distinct tissue-specific expression patterns of *QvGASTs* indicate that the functions of *QvGAST* genes have undergone tissue differentiation, suggesting functional differences in regulating the development and formation of different organs. *QvGAST* genes may be involved in various physiological processes of Chinese cork oak, including flowering development, leaf development, root growth, stem development, fruit maturation, and seed germination. Additionally, given that other species respond to GA and ABA during the process of seed germination [2,18,43], we investigated the expression patterns of *QvGAST* genes in seeds after hormonal treatments. Promoter analysis revealed the presence of numerous hormone-responsive cis-elements in the promoters of *QvGASTs*. A rich array of cis-elements associated with ABA responsiveness, auxin responsiveness, gibberellin responsiveness, and MeJA responsiveness was detected in the promoter regions of various *QvGAST* members. All *QvGAST* genes, except *QvGAST5*, possessed ABA responsiveness, whereas *QvGAST2, QvGAST3, QvGAST6, QvGAST7, QvGAST8,* and *QvGAST10* are GA responsive. Expression analysis confirmed their responsiveness to exogenous GA and ABA treatments, which was validated in qRT-PCR analysis. Interestingly, *QvGAST1* and *QvGAST7* lacked GA-responsive elements in their promoters and exhibited induced or suppressed expression patterns after GA treatment, suggesting that these *QvGAST* genes might be indirectly regulated by GA-responsive factors. Apart from *QvGAST1*, *QvGAST4*, *QvGAST5*, and *QvGAST9*, the expression patterns of the remaining genes responded to exogenous GA and ABA treatments, indicating their potential roles in integrating gibberellic acid and abscisic acid signaling pathways in cork oak. Additionally, the identification of cis-elements associated with abiotic stress responses within *QvGAST* promoters indicates their possible participation in pertinent biological mechanisms. The *GAST* gene family also plays an important role in other species, such as wheat [31], where, among the 36 *TaGASR* genes, responsive cis-regulatory elements of five important plant hormones (ABA, SA, GA, IAA, and MeJA) were identified, along with three regulatory cis-regulatory elements for abiotic stress (such as drought, low temperature, and defense).

In recent years, research has found that the GAST family plays an important role in alleviating plant seed dormancy, bud dormancy, and other physiological activities. In many plant species, seed dormancy and germination are controlled by two main plant hormones (ABA and GA) and temperature [44,45].

Subsequently, qRT-PCR analysis was performed to examine the expression of 10 *QvGAST* genes at two time points under different hormonal treatments. *QvGAST5* and *QvGAST9* were found to be non-expressing during this process. *QvGAST1*, *QvGAST2*, *QvGAST3*, and *QvGAST6* showed an increase in expression after GA_4+7_ treatment for 12 h, indicating their regulation by gibberellins. Upon FLU treatment, *QvGAST1*, *QvGAST2*, *QvGAST4*, *QvGAST6*, and *QvGAST7* showed elevated expression levels. After ABA treatment, *QvGAST3*, *QvGAST4*, *QvGAST6*, *QvGAST7*, and *QvGAST10* exhibited increased expression. Notably, *QvGAST1* and *QvGAST2* cluster together in the evolutionary tree and are likely to share analogous functionalities. In contrast, the expression patterns of *QvGAST7* and *QvGAST10* were opposite. Although different species may exhibit variations in their evolutionary pathways, such differences do not automatically lead to significant differences in the functions of the genes or traits under consideration. In Arabidopsis, for instance, *AtGASA4* promotes GA response and seed germination [46], while its homolog *AtGASA5* inhibits GA signaling and seed germination under paclobutrazol (PAC, a GA biosynthesis inhibitor) treatment [47]. Similarly, in rice, despite *OsGASA4* and *OsGASA6* having similar physiological functions, such as inducing GA and inhibiting ABA, they cluster into different subgroups [48]. We observed a significant decrease in gene expression after 24 h of gibberellin treatment compared to 12 h. These could be *GAST* genes that promote gibberellin synthesis, and as the gibberellin content increases, it inhibits its own synthesis [1]. Therefore, there may be additional mechanisms to inhibit GAST synthesis, which requires further investigation. The increase in exogenous gibberellin might lead to reduced endogenous gibberellin production, thereby resulting in a substantial decrease in gene expression.

Arabidopsis *AtGASA4*, *AtGASA5* [46], and *AtGASA6* [42], wheat *TaGASR7* and *TaGASR34* [31], and rice *OsGSR1* [32] genes play key roles in controlling seed dormancy and germination. Pear *PpyGAST1* has a promoting effect on bud dormancy release [1]. Based on the evolutionary tree of the *GAST* family members (Figure 2), we identified that some Chinese cork oak *QvGASTs* are closely related to the homologs of wheat *TaGASR34*, *TaGASR7*, rice *OsGASR1*, and Arabidopsis *AtGASA4*, *AtGASA6*, implying that they may have similar functions. Wang found that *OsGSR1* is a positive regulator of GA signaling [32]. Sun reported that GA upregulates the expression of Arabidopsis *AtGASA14* [49], while transcriptional regulatory factors inhibiting GA response downregulate the expression of *AtGASA14*. Similarly, we found that *QvGAST1* and *QvGAST2* on the same branch of the evolutionary tree are upregulated after GA treatment, showing increased sensitivity to GA, supporting the involvement of these *QvGAST* genes in GA signaling. Specifically, *QvGAST2* was significantly upregulated during the process of emergence of the seeds, leading us to speculate that *QvGAST2* might be involved in the regulation of epicotyl dormancy in Chinese cork oak. However, the biological role of *QvGAST*s in regulating epicotyl dormancy release remains largely unknown and requires further investigation in future studies.

## 5. Conclusions

This study presents the first systematic genomic analysis of the *QvGAST* gene family in cork oak. A total of 10 *QvGAST* genes were identified, enriching our understanding of the cork oak *QvGAST* gene family through further bioinformatics analysis. Tissue-specific expression analysis revealed high expression of *QvGAST3*, *QvGAST7*, and *QvGAST10* in seeds, suggesting their potential involvement in the physiological process of seed dormancy. Furthermore, hormone treatments revealed several *QvGAST* genes responsive to GA and ABA. *QvGAST1*, *QvGAST2*, *QvGAST3*, and *QvGAST6* were upregulated by gibberellin, while *QvGAST3*, *QvGAST4*, *QvGAST6*, *QvGAST7*, and *QvGAST10* showed increased expression after ABA treatment. These findings suggest the close involvement of these *QvGAST* genes in plant hormone signaling pathways, further influencing epicotyl dormancy in Chinese cork oak. *QvGAST2* was significantly upregulated during the process of releasing epicotyl dormancy in response to GA, suggesting that *QvGAST2* might be involved in the regulation of epicotyl dormancy in Chinese cork oak. These discoveries provide valuable information for further investigation into the functions of these candidate genes. This study offers new insights into the evolution of the *GAST* gene family in cork oak and the potential molecular mechanisms underlying the integration of GA biosynthesis and ABA signaling in cork oak seed dormancy release.

## Data Availability

Data are contained within the article and Supplementary Materials.

## Supplementary Materials

The following supporting information can be downloaded at: https://www.mdpi.com/article/10.3390/plants13091247/s1, Table S1: Analysis of physicochemical properties of members of GAST gene family in Chinese cork oak; Table S2: Oligonucleotide primer sequences used for qRT-PCR and in Chinese cork oak experiments.
