# Supplementary material for: Genome-Wide Identification of GAST Family Members and Their Potential Roles in Epicotyl Dormancy in Chinese Cork Oak (Quercus variabilis)
Source: Plants (Basel). 2024 Apr 30;13(9):1247. doi: 10.3390/plants13091247 (PMC11085511; doi:10.3390/plants13091247)
Supplement: Supplementary file 1 [file plants-13-01247-s001.zip › plants-2960368-supplementary.pdf]

Supplementary Information

Table S1 Analysis of physicochemical properties of members of *GAST* gene family in Chinese cork oak

Gene ID	Gene name	Number of Amino Acid/aa	Molecular Weight/KDa	Isoelectric point	Instability Index	Aliphatic Index	Grand Average of Hydropathicity
QV_Chr02G01770.1	*QvGAST1*	88	9.65	8.71	32.73	57.61	-0.080
QV_Chr02G01780.1	*QvGAST2*	88	9.63	8.71	35.45	57.61	-0.045
QV_Chr02G07160.1	*QvGAST3*	144	15.63	9.59	49.08	80.00	0.110
QV_Chr02G18590.1	*QvGAST4*	111	12.35	9.56	53.17	69.64	-0.366
QV_Chr02G34760.1	*QvGAST5*	194	20.73	9.52	76.94	49.02	-0.418
QV_Chr02G34800.1	*QvGAST6*	171	18.69	9.18	91.75	47.89	-0.602
QV_Chr03G22170.1	*QvGAST7*	105	11.62	9.32	44.22	76.00	-0.063
QV_Chr06G17900.1	*QvGAST8*	96	10.45	8.68	28.90	68.02	0.100
QV_Chr08G16600.1	*QvGAST9*	88	9.62	8.42	52.16	45.57	-0.251
QV_Chr10G05310.1	*QvGAST10*	109	12.07	8.61	46.66	74.22	-0.032

Table S2 Oligonucleotide primer sequences used for qRT-PCR and in Chinese cork oak experiments

Gene name	Forward primer	Reverse primer
QvActin7	5´ ACAGTGTCTGGATCGGAGGA 3´	5´ GGACCACTCTCGTCGTACTC 3´
QvGAST1	5´ TCCTGCTTGTTTGCCTTGTC 3´	5´ ACTTCTCACAGCAAACACCG 3´
QvGAST2	5´ AATGGCTGGTTCAAGCTTCT 3´	5´ CTCTTGCCCTTGGAGTTCTT 3´
QvGAST3	5´ CAGCCATGACCATCTCAAAG 3´	5´ ACAGGTTTGGCCTAGACGAT 3´
QvGAST4	5´ TGGATGAAGGGGAAAACGAAC 3´	5´ GCGTTCTTGTTGCCATAGGT 3´
QvGAST5	5´ TTCAGAGATGCCGCTTAGCT 3´	5´ GCACTTGTATCTTTTGCCGTG 3´
QvGAST6	5´ TCCCACTTTATGCACCACCT 3´	5´ TGGCATCTCTGATCACATCTG 3´
QvGAST7	5´ TTTGCCTTTCGTGGACTGTG 3´	5´ AGCACCTCCCACACATTTCT 3´
QvGAST8	5´ TTGTGACTCCAAATGCGGTG 3´	5´ TGCCCTTGGAGTTCAACTTG 3´
QvGAST9	5´ CAACCTATGCTGTGCGAAGT 3´	5´ TCCTCCTTCCTTAGTTTTTCCAGT 3´
QvGAST10	5´ TTGGGGGCTTTATTCTTCTG 3´	5´ AAGTTTGGCCTCGAGTGAAG 3´
